# Cost-Effectiveness of Sleeve Gastrectomy and Gastric Bypass as Revisional Surgery on Antidiabetic Reimbursement: A Nationwide Cohort Study

**DOI:** 10.1097/AS9.0000000000000420

**Published:** 2024-04-11

**Authors:** Jérémie Thereaux, Mohammed Bennani, Jean Khemis, Elisabeth Ohayon, Isabelle Visnovec Buissez, Alexandre Lafourcade, Laëtitia Quiriconi, Caroline Philippe, Jean-Michel Oppert

**Affiliations:** * From the Department of General, Digestive and Metabolic Surgery (J.T.), La Cavale Blanche University Hospital, Boulevard Tanguy Prigent, Brest, France; † University of Brest, CHU Brest, UMR 1304, Western Brittany Thrombosis Group, Brest, France; ‡ Qualees, Paris, France; § FNAMN, Cenon, France; ‖ Department of Nutrition, Pitié-Salpêtrière Hospital, Assistance Publique-Hôpitaux de Paris (AP-HP), Sorbonne University, Paris, France.

**Keywords:** antidiabetic treatments, bariatric surgery, cost-effectiveness, gastric bypass, revisional procedures, sleeve gastrectomy

## Abstract

**Objective:**

This study compared the effectiveness of 4 main revisional bariatric surgery (RBS) sequences after sleeve gastrectomy (SG) and adjustable gastric banding (AGB), on the reimbursement of antidiabetic treatments in France.

**Background:**

Few large-scale prospective cohort studies have assessed the changes in antidiabetic treatments after RBS.

**Method:**

This nationwide observational population-based cohort study analyzed data from the French National Health Insurance Database. All patients who underwent primary SG and AGB in France between January 2012 and December 2014 were included and followed up until December 31, 2020. The changes in categories and costs of reimbursed antidiabetic treatments across different RBS sequences were assessed (presented as follows: bariatric surgery (BS)-RBS).

**Results:**

Among the 107,088 patients who underwent BS, 6396 underwent RBS, 2400 SG-GBP (SG converted to gastric bypass [GBP] during follow-up), 2277 AGB-SG, 1173 AGB-GBP, and 546 SG-SG. Pre-RBS insulin was used in 10 (2.9%), 4 (0.9%), 8 (2.4%), and 10 (2.6%) patients, respectively. Two years after RBS, the treatment discontinuation or decrease (the change of treatment to a lighter one category rates [eg, insulin to bi/tritherapy]) was 47%, 47%, 49%, and 34%, respectively. Four years after RBS, the median annual cost per patient compared with baseline was lower (*P* < 0.01) for all sequences, except SG-SG (*P* = 0.24). The most notable effect concerned AGB-GBP (median of more than 220 euros to 0).

**Conclusions:**

This study demonstrated the positive impact of RBS over a 4-year follow-up period on antidiabetic treatments reimbursement, through the reduction or discontinuation of treatments and a significant decrease in costs per patient.

## Abstract 2

Mini abstract:

This real-world study based on 107,088 patients who underwent bariatric surgery (French National Health Insurance Database) demonstrated the positive impact of revisional bariatric surgery over a 4-year follow-up period on antidiabetic treatments reimbursement, through the reduction or discontinuation of treatments and a significant decrease in costs per patient.

## INTRODUCTION

Obesity is a chronic disease characterized and defined by excess body fat with deleterious health consequences and is associated with a higher risk of several comorbidities, including type 2 diabetes (T2DM). With around 30,000 to 50,000 bariatric surgery (BS) procedures performed per year in France between 2010 and 2020, the country is one of the global leaders in terms of the number of bariatric operations conducted. Sleeve gastrectomy (SG) and gastric bypass (GBP) are the most commonly performed procedures.^1,2^ The improvement or remission of T2DM, following BS, has been widely documented, in controlled clinical trials^3^ and studies using nationwide medico-administrative databases.^4^ While BS leads to sustained weight loss and improved long-term mortality rates,^5,6^ some patients develop long-term complications, especially after adjustable gastric banding^7^ (AGB) or SG^8^ (such as gastroesophageal reflux diseases), or experience weight regain and associated T2DM relapse.^4^ In some of these cases, a revisional BS (RBS) could be indicated.

The effectiveness of RBS in terms of weight loss has been demonstrated for both SG^9^ and AGB^10^ although the effect size appears to be lower than that of primary BS.^9,10^ Some studies tend to show a positive effect of RBS on the treatment of T2DM, regardless of the surgical sequence.^9,11,12^ However, this body of evidence mainly relies on studies compiling heterogeneous data or with a small sample size and would need to be strengthened.

The aim of this study, using the French national health insurance database (Système National des Donnée de Santé [SNDS]), is to assess the effectiveness of RBS after SG or AGB on T2DM by analyzing the changes in antidiabetic treatment categories and the economic impact of RBS on antidiabetic treatment reimbursements.

## MATERIAL AND METHODS

### Data Sources

This is a retrospective cohort study based on the SNDS data that integrated the main French national health databases. The SNDS contains individual anonymized information on all public or private practices, medical and paramedical encounters, drugs claims, hospital admissions and procedures, and dates of death. These data were linked to the creation of a longitudinal record of outpatient health encounters, hospital diagnoses, and drug dispensing for more than 99% of the French population from birth to death. France’s health insurance system combines public and private/mutual complementary insurance to ensure universal coverage, including mandatory public health insurance for most residents and additional schemes for low-income individuals. The SNDS is one of the largest databases of its kind in the world, allowing for systematic follow-up of all medical care received in France, including low-income people. The evidence obtained from such data has been extensively used to guide the public health policies in France. Our team previously published results in the field of BS using the SNDS.^2,4,5,13^

Analyses of the SNDS database for this study received the ethical and methodological approval of the French Health Data Hub (Entrepot des données de santé) (N° T53918201804270) and the French Expertise Committee for Research, Studies, and Evaluations in the field of Health (Comité d’Expertise pour les Recherches, les Études et les Évaluations dans le domaine de la Santé). This study was authorized by the French Personal Data Protection Authority (Commission Nationale Informatique et Libertés, authorization number: 918424). Informed patient consent was not required because the data included in the SNDS were anonymized, and all analyses were performed on the dedicated secured portal of the French National Health Insurance.

### Study Design and Population

This retrospective national cohort study targeted all obese patients in the SNDS database with an obesity diagnosis who underwent BS, including SG, AGB, and GBP, for the first time between January 2012 and December 2014. All patients who underwent RBS with SG or AGB as the primary procedure were followed until December 2020. Patients who underwent other procedures, such as the placement of an intragastric balloon, subcutaneous implantation of a gastric stimulator with the placement of a gastric parietal tube, laparoscopy, procedures corresponding to the removal of a gastric band alone, or RBS, within the first year after primary BS were excluded. In our study, we chose to exclude primary GBP because there is no specific code in the French Surgical Classification describing the revision procedure after GBP.

The French-specific disease codes and International Classification of Diseases, 10th Revision (ICD-10) codes for obesity used to extract the patient data have been described previously.^2,4,5^

### Data Collection

The demographic and basic clinical characteristics included sex, age, type of bariatric procedure (AGB, SG, and GBP), and body mass index (BMI) categories (25–29.9, 30–39.9, 40–49.9, 50 or more, kg/m²) at the first BS and, when applicable, at the second BS. Changes in the BMI were determined by the switching of categories before BS and before RBS.

Low-income families were identified based on the presence of universal health insurance coverage (Couverture Maladie Universelle). Patients with diabetes, hypertension, and hyperlipidemia were defined as those who received 1 of the 3 corresponding drug reimbursements during the year before the revision surgery. Three categories of antidiabetic treatments were defined depending on reimbursement: insulin use, monotherapy, and bi/tritherapy (for oral agents). These categories permit the observation of changes in treatments after RBS, discontinuation if the treatment is stopped, and decrease if the patients switch to a lighter treatment (eg, insulin use to bi/tritherapy or bi/tritherapy to monotherapy) or increase (eg, monotherapy to bi/tritherapy). Patients with obstructive sleep apnea were defined as those who had received reimbursement for continuous positive airway pressure during the previous year. ICD-10 codes and the Anatomical Therapeutic Chemical code used for data collection were as previously detailed.^2,4,13^

### Statistical Analyses

Statistical analyses were performed using SAS software (version 9.4; SAS Institute, Cary, NC). Descriptive analyses of qualitative and ordinal variables included the frequency and percentage of each item, and quantitative variables included the number of patients, mean and confidence interval, SD, median, and interquartile range (IQR). Categorical data were presented as counts and percentages. We used Fisher exact tests for categorical variables and Student *t* tests or nonparametric tests (Mann–Whitney *U* and Kruskal–Wallis tests) for numerical variables, as appropriate. The significance threshold was set at *P* = 0.05.

## RESULTS

### Flow Chart of the Study

**FIGURE 1.**
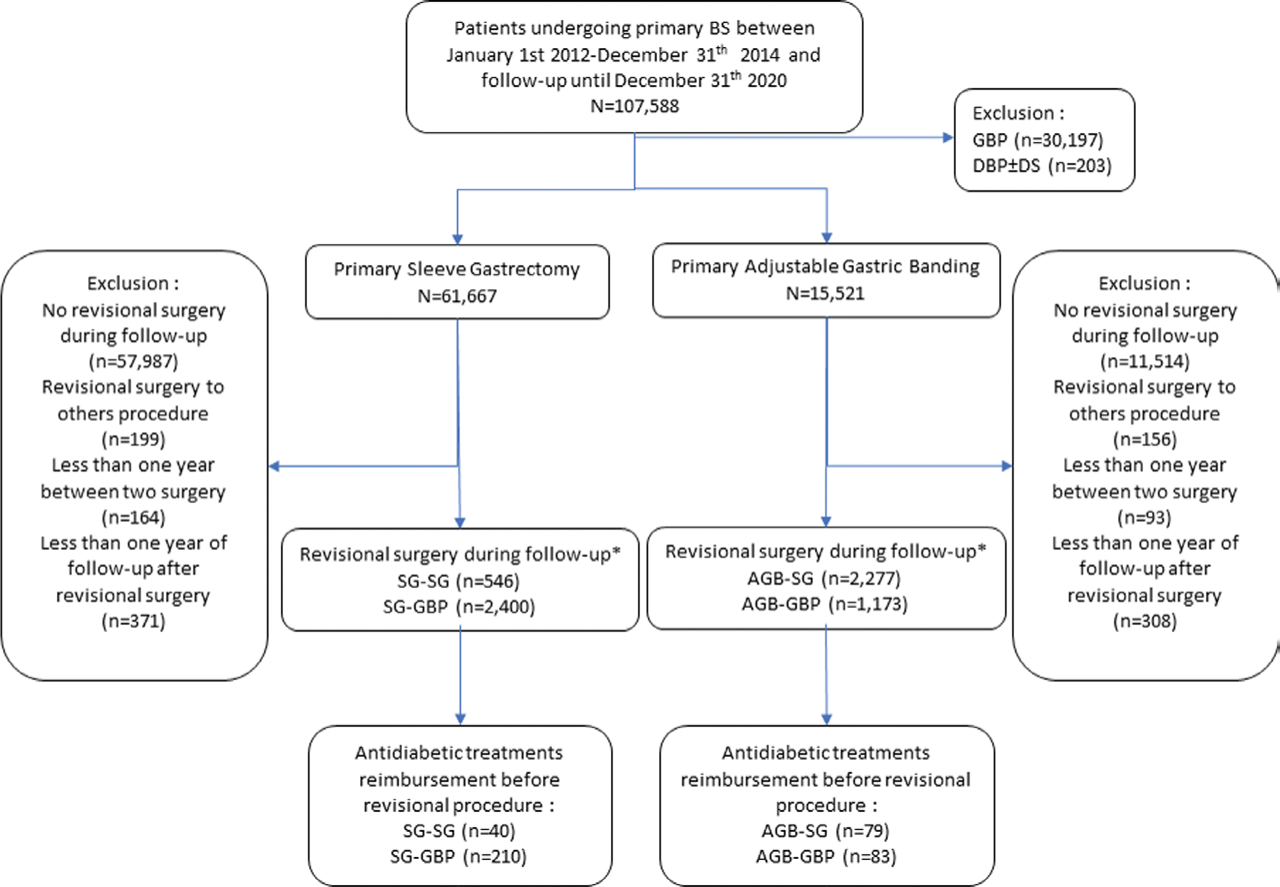
Flow chart. *Sequence of BS should be reading as primary BS to revisional BS; for example, SG-GBP means primary SG converted to GBP during follow-up. BPD±DS, biliopancreatic derivation ± duodenal switch.

The study population was drawn from an initial sample of 107,588 patients with a primary BS treated in 2012, 2013, and 2014 (Fig. 1). Among these patients, 61,667 (57%) patients underwent SG and 15,521 (14%) patients underwent AGB, as the first bariatric intervention.

Among these patients, 6396 (8.3%) patients required RBS, and 4 surgical sequences were identified and termed as “initial surgery”-“revisional surgery”: 2400 (37.5%) patients had SG-GBP, 2277 (35.6%) patients had AGB-SG, 1173 (18.3%) patients had AGB-GBP, and 546 (8.6%) patients had SG-SG (Fig. 1).

### Characteristics of Patients at the Time of RBS

Table 1 describes the baseline characteristics of the patients at the time of primary BS and RBS, including reimbursements for the treatment of coexisting conditions. Among patients undergoing RBS, considering sequences SG-GBP, AGB-SG, AGB-GBP, and SG-SG, the BMI categories were lower at the time of RBS compared with primary BS, respectively, by 46.4%, 23.7%, 23.7%, and 43.8%. The median (months) interval between the primary surgery and RBS ranged from 46.1 (IQR: 32.2–60.7) for SG-SG to 49.4 (IQR: 35.2–63.5) for AGB-GBP. The patient rates for antidiabetic treatments reimbursed in the year preceding RBS ranged from 3.5% for AGB-SG to 8.8% for SG-GBP.

**TABLE 1.** Baseline Characteristics of Patients Undergoing Primary Bariatric Surgery (Sleeve Gastrectomy or Adjustable Gastric Banding) During the Years 2012 to 2014 and Revisional Bariatric Surgery (n = 6396) During the Years 2013 to 2020, the SNDS Data*

Characteristics	Sequence of Revisional Bariatric Surgery†			
	SG-GBP n = 2400	AGB-SG n = 2277	AGB-GBP n = 1173	SG-SG n = 546
Age (yr) at the time of BS	39.4 (11.5)	33.2 (10.8)	35.9 (10.9)	37.4 (11.3)
Age (yr) at the time of RBS	43.5 (11.4)	37.3 (10.9)	40.0 (11.0)	41.3 (11.2)
Female	2080 (86.7)	2043 (89.7)	1049 (89.4)	457 (83.7)
Universal health insurance coverage for patients with low income	851 (35.5)	900 (39.5)	374 (31.9)	207 (37.9)
Interval (mo) between BS and RBS (median [IQR])	48.5 (33.8–62.6)	48.0 (34.0–63.0)	49.4 (35.2–63.5)	46.1 (32.2–60.7)
BMI categories (kg/m^2^) at time of BS‡				
25.0–29.9	2 (0.1)	0	0	0
30.0–39.9	564 (23.5)	949 (41.7)	435 (37.1)	122 (22.3)
40.0–49.9	1348 (56.2)	1191 (52.3)	664 (56.6)	343 (62.8)
>50.0	475 (19.8)	130 (5.7)	70 (6.0)	79 (14.5)
Obesity, BMI unspecified	11 (0.5)	7 (0.3)	4 (0.3)	2 (0.4)
BMI categories (kg/m^2^) at time of RBS‡				
25.0–29.9	55 (2.5)	12 (0.5)	5 (0.4)	8 (1.5)
30.0–39.9	1199 (54.2)	1169 (51.7)	544 (46.6)	283 (53.7)
40.0–49.9	796 (36.0)	955 (42.2)	547 (46.8)	206 (39.1)
>50.0	150 (6.8)	119 (5.3)	67 (5.7)	24 (4.6)
Obesity, BMI unspecified	11 (0.5)	6 (0.3)	5 (0.4)	6 (1.1)
BMI categories at the time of RBS compared with BS				
Lower	1114 (46.4)	540 (23.7)	278 (23.7)	239 (43.8)
Higher	97 (4.0)	292 (12.8)	155 (13.2)	18 (3.3)
No change	979 (40.8)	1416 (62.2)	726 (61.9)	262 (48.0)
Undefined	210 (8.8)	29 (1.3)	14 (1.2)	27 (4.8)
Antihypertensive treatment at the time of RBS§	569 (23.7)	295 (13.0)	224 (19.1)	112 (20.5)
Lipid-lowering treatment at the time of RBS§	235 (9.8)	83 (3.6)	77 (6.6)	43 (7.9)
Obstructive sleep apnea syndrome treatment at the time of RBS§	365 (15.2)	283 (12.4)	182 (15.5)	70 (12.8)
Antidiabetic treatment at the time of RBS§	210 (8.8)	79 (3.5)	83 (7.1)	40 (7.3)
Antidiabetic treatment categories and annual cost at the time of RBS	210	79	83	40
Monotherapy	82 (39.0)	34 (43.0)	33 (39.8)	17 (42.5)
Bi/tritherapy	67 (31.9)	26 (32.9)	25 (30.1)	13 (32.5)
Insulin use	61 (29.0)	19 (24.1)	25 (30.1)	10 (25.0)
Median annual Cost of antidiabetic treatment reimbursement per patient (euros)	310.6 (65.7–835.8)	180.1 (45.6–734.0)	241.3 (53.7–731.7)	122.4 (57.2–583.1)

* Values are expressed as mean (SD), median (IQR), or numbers (%), as appropriate. Patients undergoing RBS in the year following primary BS were excluded (n = 936).

† Sequence of BS should be reading as BS to RBS; for example, SG-GBP means primary SG converted to GBP during follow-up.

‡ Body mass index is the weight in kilograms divided by the square of the height in meters.

§ Defined as at least 3 reimbursements during the past year before RBS.

### Evolution of Antidiabetic Treatment Categories After RBS

**FIGURE 2.**
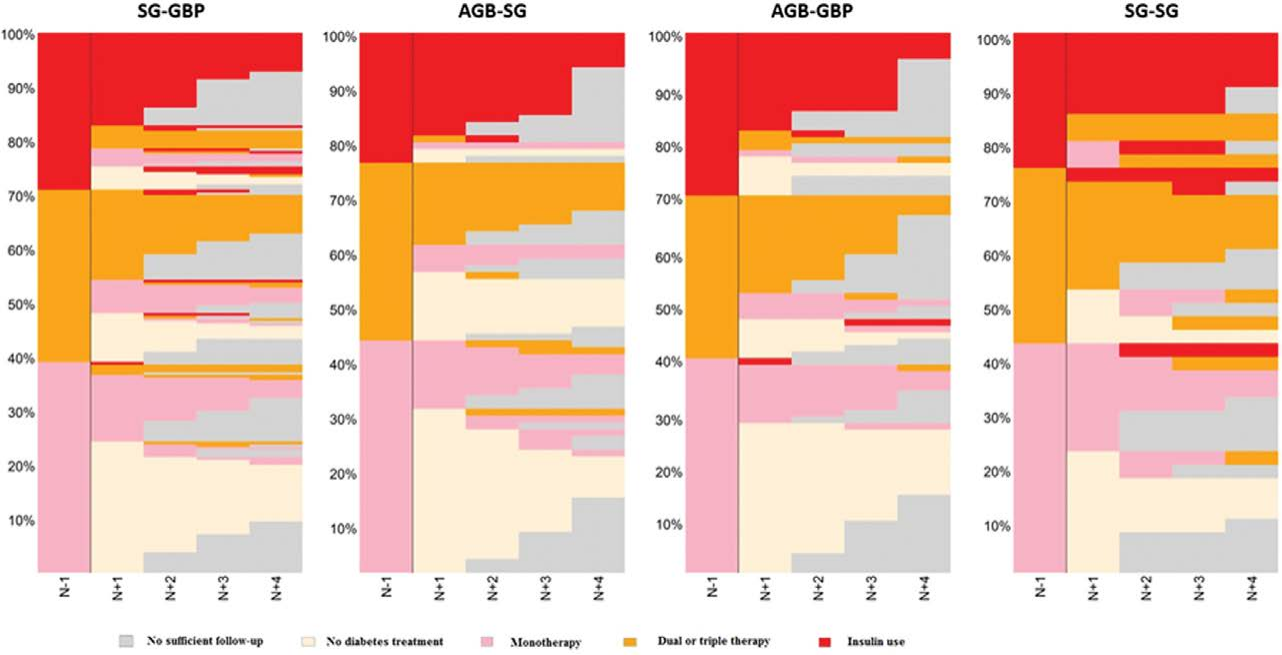
Course of care (sequence charts) of patients with antidiabetic treatments (continuation, discontinuation, or relapse) reimbursed 1 year before revisional bariatric surgery* (n = 481) and followed up until December 31, 2020. *Sequence of BS should be reading as primary BS to revisional BS; for example, SG-GBP means primary SG converted to GBP during follow-up.

Of the 412 patients who received antidiabetic treatment at the time of the RBS, 115 patients (27.9%) were treated with insulin. Two years after RBS, considering the categories SG-GBP, AGB-SG, AGB-GBP, and SG-SG, the rates of discontinuation or decreases in the categories of reimbursement were 47.0%, 47.1%, 49.3%, and 34.4%, respectively. Four years after RBS, the rates of reimbursement discontinuation and decreased categories of treatment were 46.1%, 45.9%, 56.6%, and 30.4%, respectively (Table 2). Figure 2 shows the course of care (sequence charts) of patients receiving antidiabetic treatments (continuation, discontinuation, or relapse) reimbursed 1 year before RBS (n = 412) and followed up until December 31, 2020. For example, in the AGB-SG sequence, approximately 27% of patients received insulin reimbursement 1 year before RBS and 17% 1 year later. No de novo insulin reimbursement occurred in the other categories until 4 years of follow-up.

**TABLE 2.** Evolution of Antidiabetic Treatment Reimbursement and Cost in Diabetic Patients Undergoing RBS, the SNDS Data*

Evolution of Antidiabetic Treatment Categories During Follow-Up After RBS	Sequence of Revisional Bariatric Surgery†				*P* ‡
	SG-GBP	AGB-SG	AGB-GBP	SG-SG	
At year 2 (n = data available)	170	68	67	32	
Discontinuation of reimbursement	56 (32.9)	28 (41.2)	27 (40.3)	6 (18.8)	
Decrease (but no discontinuation)	24 (14.1)	4 (5.9)	6 (9.0)	5 (15.6)	
Increase	9 (5.3)	2 (2.9)	0	2 (6.3)	
No change	81 (47.6)	34 (50.0)	34 (50.7)	19 (59.4)	
Median annual cost of antidiabetic treatment reimbursement per patient (euros)	54.7 (0.0–337.8)	6.1 (0.0–141.1)	0.0 (0.0–61.0)	61.8 (0.0–264.6)	<0.01; <0.01; <0.01; <0.01
At year 4 (n = data available)	104	37	30	23	
Discontinuation of reimbursement	30 (28.8)	14 (37.8)	13 (43.3)	4 (17.4)	
Decrease (but no discontinuation)	18 (17.3)	3 (8.1)	4 (13.3)	3 (13.0)	
Increase	7 (6.7)	2 (5.4)	2 (6.7)	4 (17.4)	
No change	49 (47.1)	18 (48.6)	11 (36.7)	12 (52.2)	
Median annual cost of antidiabetic treatment reimbursement per patient (euros)	60.7 (0.0–337.7)	0.0 (0.0–86.9)	0.0 (0.0–45.6)	66.2 (6.3–375.6)	<0.01; <0.01; <0.01; NS

* Values are expressed as median (IQR) or numbers (%), as appropriate.

† Sequence of BS should be reading as BS to RBS; for example, SG-GBP means SG converted to GBP during follow-up.

‡ For comparison of cost of antidiabetic treatment for each sequence, *P* value is compared with baseline annual cost before RBS in each category.

NS indicates non significant.

### Economic Impact of RBS on Reimbursement of Antidiabetic Treatments

**FIGURE 3.**
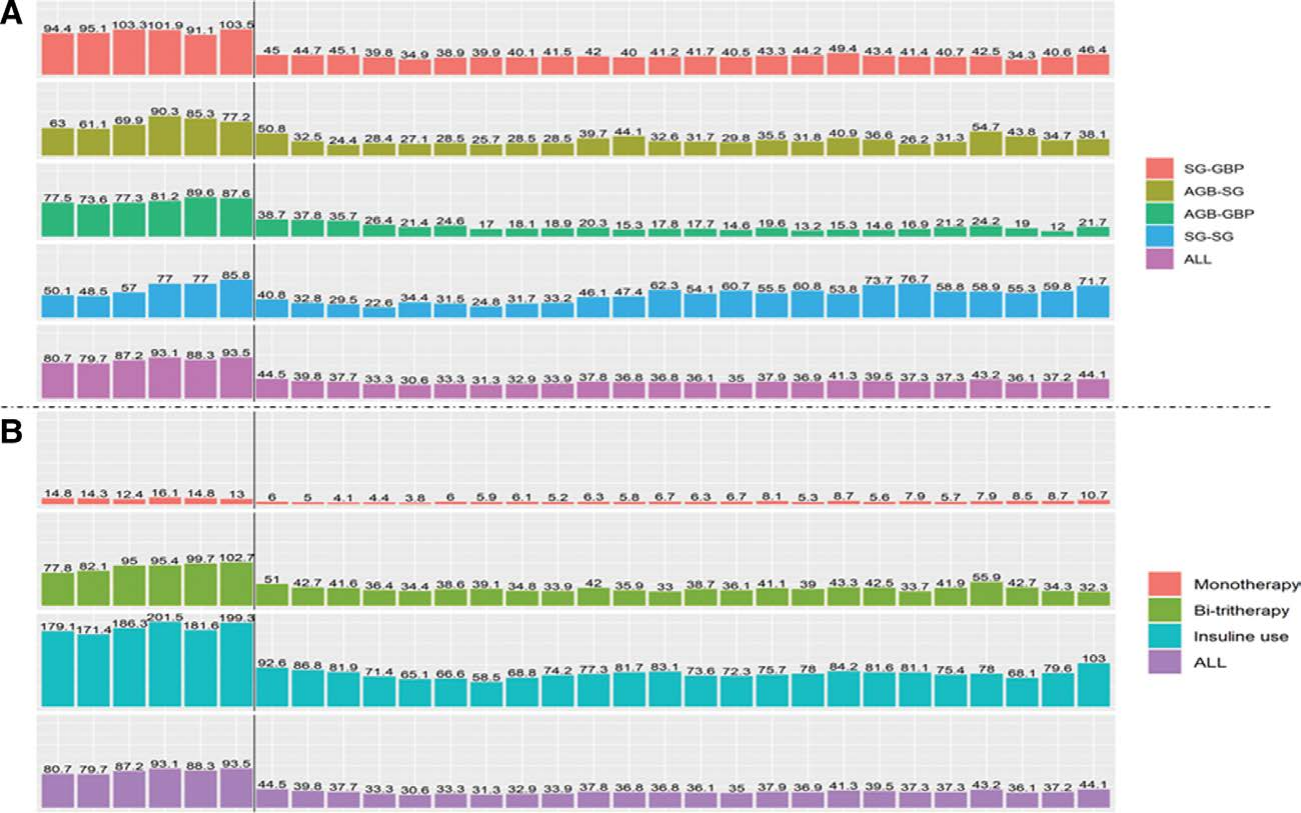
The mean cost (euros) of antidiabetic treatments reimbursed per patient by a 2-month period in patients with antidiabetic treatments reimbursed 1 year before revisional bariatric surgery* (n = 481) and followed up until December 31, 2020. *Sequence of BS should be reading as primary BS to revisional BS; for example, SG-GBP means primary SG converted to GBP during follow-up. Black vertical bars indicate the time of revisional BS.

Figure 3 shows the mean cost (euros) of antidiabetic treatments reimbursed per patient over a 2-month period in patients with antidiabetic treatments reimbursed 1 year before RBS and followed up until December 31, 2020, depending on the sequences and treatment categories. At 4 years, the annual median cost (euros) per patient compared with the baseline pre-RBS was lower (*P* < 0.01) for all sequences (SG-GBP, AGB-SG, and AGB-GBP), except for SG-SG (*P* = 0.24), respectively: 310.6 (65.7– 835.8) versus 60.7 (0.0–337.7); 180.1 (45.6–734.0) versus 0.0 (0.0–86.9); 241.3 (53.7–731.7) versus 0.0 (0.0–45.6); and 122.4 (57.2–583.1) versus 66.2 (6.3–375.6) (Table 2). At 4 years, the annual median cost (euros) per patient was lower than that at baseline (*P* < 0.01) for all antidiabetic treatment categories at the time of revisional surgery (bi/tritherapy and insulin), except for monotherapy (*P* > 0.05).

## DISCUSSION

The positive impact of BS on T2DM has been demonstrated by the results of long-term randomized controlled trials and analyses of nationwide databases such as the one studied here.^3,4^ However, few studies have extensively assessed the impact of RBS on the treatment of patients with T2DM, especially based on a nationwide database.^9,11^ Using the French medico-administrative SNDS database and focusing on reimbursement for antidiabetic treatment, we found that RBS (after AGB or SG) resulted in a substantial decrease in the number of prescribed antidiabetic treatments and the associated reimbursement costs. The data from this study strongly suggest that RBS has a favorable effect on antidiabetic treatments immediately after the procedure and over the course of the following 4 years.

RBS is increasingly becoming a public health and economic concern because of the major growth in primary BS performed in several countries over the past 2 decades.^1^ The most frequent indications of RBS, as shown by a cohort of expert centers including more than 3000 patients, are weight regain/weight loss failure and gastroesophageal reflux disease.^14^ Among the studies based on large databases,^14,15^ Dang et al^15^ showed in their metabolic and bariatric surgery accreditation and quality improvement program database that the SG-GBP sequence (n = 13,432) provides more complications compared with primary GBP. They found a lower proportion of patients with T2DM (30% vs 11%) compared with primary GBP at baseline before RBS. Our rate of 10% of antidiabetic treatments before RBS in the SG-GBP sequence is, therefore, in line with that of a previous US study.^15^ However, this study did not provide outcomes on coexisting conditions. Most studies assessing RBS focused only on postoperative morbi-mortality.^15,16^ The literature review conducted by Yan et al^17^ from a selection of 30 articles showed residual diabetes in 14% to 38% of patients at the time of RBS and improvement of diabetes in 65% to 100% of patients. In our study, there is a “drop” in the treatment determined by a shift from one treatment group to another, for example, a subject on bi/tritherapy before the intervention who would be on monotherapy after the intervention, a subject on insulin who would switch to bi/tritherapy, or treatment cessation determined by taking less than 3 antidiabetic treatments or stopping treatments within 1 year after the intervention. In addition, we studied the entire French population who underwent an initial bariatric procedure between 2012 and 2014, followed by a revision procedure within 8 years (until December 2020). As described previously,^11,17^ a surgical sequence with GBP in the second intervention yielded higher improvement in diabetes after AGB than after SG, which was consistent with the findings of our study; however, our rate was lower than that reported in this review (65% of diabetes improvement with AGB-GBP in our study vs 79% in the review^17^). This can be explained by the population size of this study. Another critical issue was that the median time between primary and RBS was more than 4 years in our nationwide study. These data explain why it is difficult to determine the mid- to long-term outcomes of RBS in patients with T2DM.

Although several published^18,19^ analyses have demonstrated the cost-effectiveness of BS in patients with obesity and T2DM, none have addressed the impact of RBS on the reimbursement of antidiabetic treatments. We know that SG and GBP, as initial BS, have a stronger impact on the reimbursement of antidiabetic treatments than AGB.^4^ Here, the cost of antidiabetic treatments is divided by 2 as early as the next 2 months following RBS. The most impressive effect was found for the AGB-GBP sequence, where the annual cost per patient shifted from a median of more than 220 euros to 0 after 4 years. We cannot compare our outcomes to literature. Our sequence chart graphics describe in an innovative way, and for the first time, in a cohort of patients undergoing RBS, the impact of the RBS on antidiabetic treatment category evolution within 4 postoperative years after RBS, depending on the surgical sequence that was followed. However, unlike the initial BS, this benefit seems to be reduced during the 4 years of follow-up, especially for the SG-SG sequence. Even though SG-SG has been found recently to provide equivalent long-term outcomes than primary SG, it is well known that SG is not as effective as GBP for the improvement/remission of metabolic coexisting conditions, such as T2DM.^4,12^ Regardless of the surgical sequence, the cost of monotherapy after revisional surgery presented the smallest decrease over time.

The major strength of this study lies in the innovative way in which the cost reimbursement of antidiabetic treatment per patient over time, in a real population, was presented and discussed. This is the first large-scale study on the mid-term outcomes of this critical topic. The data presentation permitted the assessment of cost evolution depending on RBS sequences using the entire French population. The other strengths of using the SNDS with outpatient and inpatient data include the comprehensive record of data, minimum loss to follow-up, and absence of exclusion of socioeconomically disadvantaged patients, as detailed previously.^4,5,13^ However, this study has a few limitations. Patients with diabetes were defined based on the dispensing of antidiabetic treatments, and the evolution of their diabetic status was defined based on these treatments. The precise data on glycemic control were not available in the SNDS database. Although the initial sample based on the entire French population was large, the subgroups analyzed here were rather small, especially due to the data available for follow-up (median time between primary and RBS >4 years). Hence, it was not possible to perform a multivariate analysis to better explore the optimal choice of revision procedures in cases of persistent T2DM after primary BS. Using ICD-10 codes to describe the severity of obesity is another limitation of our study because the range of ICD-10 nomenclature of 10 kg/m² does not allow the precise description of the weight evolution of patients in a relevant manner. Weight regain management after GBP failure is a critical issue, and no clear decision algorithm has been established by Western scientific societies. Generally, banding the gastric pouch or shortening the common limb is often cited.^20,21^ We chose to exclude primary GBP in our study because there is no specific code in the French Surgical Classification describing the revision procedure after GBP.

This study shows a large positive effect of RBS on diabetes through the reduction or cessation of treatments in more than half of the patients with diabetes and by a significant reduction in the costs of reimbursement of diabetes treatments during the 4 years following the intervention, especially in the AGB-GBP sequence. In some cases, the cost was cut by 2 as early as 2 months after revisional surgery. A longer follow-up period is needed to better assess the long-term outcomes and confirm the long-term cost-effectiveness of RBS for antidiabetic treatment.

## ACKNOWLEDGMENTS

Participated in research design: J.T., M.B., J.K., E.O., I.V.B., C.P., J.-M.O.; participated in the writing of the paper: J.T., M.B., L.Q., J.K., E.O., I.V.B., C.P., J.-M.O.; participated in the performance of the research: J.T., M.B., L.Q., J.K., E.O., I.V.B., C.P., J.-M.O., A.L.; contributed new reagents or analytic tools: A.L., J.T., J.-M.O.; participated in data analysis: A.L., J.T., J.-M.O., L.Q., M.B.
